# A bird's eye view: using geographic analysis to evaluate the representativeness of corvid indicators for West Nile virus surveillance

**DOI:** 10.1186/1476-072X-6-3

**Published:** 2007-01-30

**Authors:** Samara T David, Sunny Mak, Laura MacDougall, Murray Fyfe

**Affiliations:** 1 Canadian Field Epidemiology Program, Public Health Agency of Canada, Ottawa, Canada; 2 Epidemiology Services, British Columbia Centre for Disease Control, 655 West 12^th ^Avenue, Vancouver, British Columbia, V5Z 4R4, Canada; 3 Vancouver Island Health Authority, 430 1900 Richmond Avenue, Victoria, British Columbia, V8R 4R2, Canada; 4 Department of Health Care and Epidemiology, University of British Columbia, Vancouver, Canada

## Abstract

**Background:**

The objective of this evaluation was to determine whether reports of dead corvid sightings and submissions of dead corvids for West Nile virus testing were representative of true corvid mortality in British Columbia in 2004, a year with no West Nile virus activity, in order to ensure the system was accurately describing corvid mortality rather than reflecting regional differences in surveillance methods.

**Results:**

Local Health Areas reported 0–159 (median = 3) dead corvid sightings and 0–209 (median = 5) submissions for West Nile virus testing. The expected numbers of dead corvid sightings and submissions for testing from each Local Health Area were 0–232 (median = 3) and 0–258 (median = 4), respectively. Twelve Local Health Areas reported significantly fewer sightings than expected; 21 reported significantly more. Eleven Local Health Areas submitted significantly fewer corvids than expected; 26 submitted significantly more.

**Conclusion:**

Some Local Health Areas were over-represented and others under-represented in terms of corvid West Nile virus surveillance indicators. Recommendations were made to improve the representativeness of corvid surveillance data. Geographic analysis can be used to evaluate the representativeness of surveillance systems and result in improvements to surveillance.

## Background

Although it is well established in Africa, Europe, the Middle East and India,[1] West Nile virus (WNv) is an emerging zoonosis in North America. Between 1999 and 2003, WNv made its way from New York City across most of North America, including human cases in the Canadian provinces of Quebec, Ontario, Manitoba, Saskatchewan, and Alberta[2]. As jurisdictions adjacent to British Columbia (BC) experienced WNv activity in 2003 and earlier years,[2,3] WNv was anticipated to arrive in British Columbia (BC) in 2004.

West Nile viral amplification occurs in a bird-mosquito-bird cycle before bridge vector mosquitoes (mosquitoes that bite both birds and mammals) infect human populations[4]. Although many bird species become infected with WNv, WNv is particularly fatal to the corvid family of birds (corvid species include crows, ravens, jays, magpies, and nutcrackers). Surveillance in areas experiencing outbreaks found that a dramatic increase in corvid mortality preceded human illness by two to six weeks[5,6]. Their high mortality rate, large size, and distinctive colouring make dead corvid sightings a good indicator for WNv activity in the ecosystem. In addition, corvid carcasses can be easily tested for WNv infection[7].

In 2004, WNv surveillance in BC involved testing dead corvids, mosquito pools, blood/organ donors, and symptomatic humans for WNv and the public using an internet-based form to report dead corvid sightings[8]. Generally dead corvid sightings reported via the internet-based form are different carcasses than those submitted for testing, with occasional overlap. The methods for collecting carcasses for testing vary regionally, with some regions hiring specific staff to collect carcasses and other regions collaborating with wildlife agencies. No evidence of WNv activity was observed in BC in 2004.

If WNv infection is confirmed in a corvid in BC, resulting public health actions include reinforcing public education around personal protection measures, and possibly heightening mosquito surveillance, initiating larviciding (if not already initiated) and/or considering the use of adulticides (if indicators provide evidence of an imminent outbreak)[9].

The objectives of this evaluation were to determine whether, in the absence of WNv, the numbers of dead corvids sighted and tested for WNv in 2004 were representative of the true corvid mortality. This evaluation examined whether the surveillance indicators demonstrated what was truly happening in the corvid populations, rather than reflecting differences in regional surveillance methods; that is, whether areas of the province that were expected to have larger numbers of dead corvids observed were reporting more dead corvid sightings and submitting more corvids for WNv testing than areas expected to have smaller numbers of dead corvids observed. Corvid surveillance could then be strengthened by addressing differences in regional surveillance systems that cause certain areas to be over- or under-represented.

## Results

Surveillance activities in 2004 occurred over a 26 week period (May 1-October 31). The public reported 1,292 dead corvid sightings. The number reported in each Local Health Area (LHA) ranged from 0 to 159 (median = 3) [see Additional file 1]. LHAs submitted 1,437 corvid carcasses for WNv testing. The number submitted by each LHA ranged from 0 to 209 (median = 5). These 1,437 submissions included 1,293 Crows (American or Northwestern), 43 Common Ravens, one Gray Jay, 28 Steller's Jays, three Blue Jays, one Clark's Nutcracker and 68 Black-billed Magpies.

The estimates of relative corvid density for each LHA ranged from 0.6 to 61.0. The number of corvid sightings expected in each LHA ranged from 0 to 232 (median = 3). The expected number of corvids tested in each LHA ranged from 0 to 258 (median = 4).

**Figure 1:**
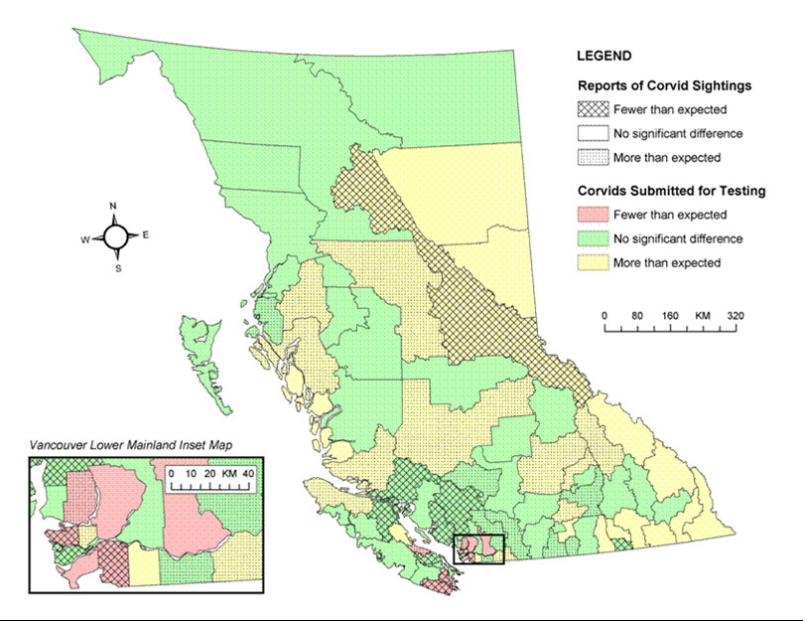
Dead corvid sightings and carcasses tested for West Nile virus versus expected, by Local Health Area.

Twelve LHAs had significantly fewer reports of dead corvid sightings than expected; 21 had significantly more (Figure 1). Eleven LHAs submitted significantly fewer corvid carcasses for WNv testing than expected; 26 submitted significantly more.

The cross-tabulation of the sighting and submission evaluations is outlined in Table 1.

**Table 1:** Cross-tabulation of corvid sighting and corvid submission results

		**Number of Local Health Areas submitting corvids for West Nile virus testing**		
		**Significantly fewer than expected**	**No significant difference from expected**	**Significantly more than expected**

**Number of Local Health Areas with public reports of dead corvid sightings**	**Significantly fewer than expected**	5	6	1
	
	**No significant difference from expected**	4	32	14
	
	**Significantly more than expected**	2	8	11

Statistical significance was determined using the exact Poisson distribution and a significance level of 0.05.

## Discussion

This geography-based evaluation demonstrated that some LHAs were sighting or testing more corvids than expected, and some were sighting or testing fewer than expected. Since WNv was not present in the province, this variation was not the result of differences in corvid mortality due to the virus, but most likely differences in the operation of regional surveillance programs or regional variations in other causes of corvid mortality.

At the time of this evaluation, there were no standards for the number of reports of dead corvid sightings or the number of corvids that should be tested in order to identify WNv in an area. We compared the numbers of corvids sighted and tested in each LHA to an expected number that was based on the proportion of all BC corvids expected in each LHA (assuming constant corvid mortality across LHAs). The absolute number of corvids sighted or tested was, therefore, not evaluated; the proportion of corvids in a particular area relative to sightings or submissions from other parts of the province was. For corvid data to be representative, the proportion sighted or submitted by each LHA should reflect the proportion expected from that LHA.

Sixty-five (78%) LHAs met or exceeded expectations for reports of dead corvid sightings and submissions of corvid carcasses for WNv testing.

Only 5 (6%) LHAs were below expectations for both indicators – Surrey, Vancouver, Cowichan, Greater Victoria, and Sooke. Three of these LHAs (Cowichan, Greater Victoria, and Sooke) were on Vancouver Island. When the two indicators were examined separately, five of the 12 (42%) LHAs reporting fewer dead corvid sightings than expected and five of the 11 (45%) LHAs submitting fewer corvid carcasses for WNv testing than expected were on Vancouver Island. To place this in context, Vancouver Island has 16 LHAs (17% of all LHAs). This lower level of surveillance activity on Vancouver Island may have been a result of lower public health emphasis or low perception of risk. Vancouver Island had been projected to be a low risk area for the initial introduction of WNv into the province.

Surrey and Vancouver, the other two LHAs that were below expectations for both corvid indicators, were both in the Vancouver Lower Mainland. In fact, six of the 11 (55%) LHAs submitting fewer carcasses for WNv testing than expected were in the Vancouver Lower Mainland. The below-expected results for these areas are most likely an artefact of over-estimation of the expected values. The calculation of the expected proportion of corvids in each LHA was based on a number of assumptions and modelling. The number of dead corvids sighted is dependent on the size of the area, the corvid density, and the human population density; however, the exact properties of these relationships are unknown. We assumed a multiplicative relationship between these three factors. As a result, this model may have resulted in erroneous classification of LHAs. The contribution of population density to determining the expected number of corvids for LHAs with high population densities (such as Vancouver and Surrey) may have resulted in over-estimates of expected values. Both Vancouver and Surrey had high raw numbers of reports of dead corvid sightings (139 and 80, respectively) and high raw numbers of corvids tested for WNv (209 and 97, respectively). This equates to more than five corvids submitted for testing per week from each of these LHAs and may have been sufficient to detect WNv activity if present. The threshold value of required corvids is unknown.

The majority of the LHAs testing more corvids than expected were in the eastern and south-eastern parts of the province. WNv was expected to enter the province from these areas since they are adjacent to Alberta, Montana, Idaho, and Washington, which reported WNv activity in 2004 and prior years. Due to low human population and corvid densities in some of these areas, expected corvid sightings and submissions were low (1–5 corvids sighted or tested per year). This small number of corvids may not be sufficient for WNv detection when the virus is introduced. Rural areas with low population and corvid densities may need to focus on other methods of WNv detection.

There is a paucity of data on the distribution of corvids in BC. An examination of the raw North American Breeding Bird Survey (BBS) point data shows that the BBS data have relatively low numbers of observation points and under-represent the northern parts of province[10]. Therefore, this model was more robust for southerly regions of the province where there was a higher density of observation points.

We used the mean number of total corvids observed in the 1994–2003 BBS bird abundance map data to estimate corvid density in each LHA. Combining the corvid species provided larger, more robust estimates for the model. Similarly, using the mean of a number of years of observations provided more stable numbers. Combining corvid species and using the 10 year bird abundance data may have limited the model's ability to account for the spatial and temporal effects of more recent fluctuations in corvid populations. Furthermore, the assumption of constant corvid mortality across the province may have been erroneous; however, it was not possible to obtain data on local corvid mortality rates.

## Conclusion

In conclusion, some LHAs were over-represented and others under-represented in BC's 2004 WNv corvid surveillance data. In addition, some data that were "representative" may not necessarily be useful (e.g., corvid surveillance data in areas with low corvid and human population densities).

To improve the representativeness of corvid surveillance data:

1. LHAs reporting fewer dead corvid sightings than expected should strive to increase reporting by the public by emphasizing this aspect of surveillance in media communications.

2. LHAs testing fewer corvids for WNv than expected should strive to increase the number of corvids submitted for WNv testing relative to other areas of the province, although an absolute number may be adequate to detect WNv presence in an area.

3. Areas with low population and corvid densities may choose to focus surveillance efforts on mosquito and human surveillance or active corvid surveillance rather than passive corvid surveillance.

4. Areas with very large population densities should not be too concerned about achieving expected values for corvid sightings and submissions as assumptions in the model used to calculate the expected values may lead to unreasonably large expected values.

The results of this evaluation and the recommendations were shared with the public health stakeholders in the spring of 2004. During the 2005 WNv surveillance season, notable increases in corvid submissions occurred on Vancouver Island, compared with previous years[11]. In addition, testing of migrating wild birds was conducted in south-eastern and coastal BC by the Canadian Wildlife Service in 2005 to assist with WNv detection in areas with low corvid densities. Regions in south-east BC also increased the number of mosquito traps in areas with low overall corvid submissions.

This evaluation demonstrates that geographic analysis can be used to evaluate the representativeness of surveillance systems and result in improvements to surveillance.

## Methods

The number of dead corvid sightings reported by the public in each of BC's 83 LHAs and the number of corvid carcasses submitted by each LHA for WNv testing were compared to the numbers expected from each LHA. The expected numbers of corvids sighted and tested from each LHA were projected to be proportional to the geographic size of the LHA, controlling for the relative corvid density and human population density (to account for the chances of a human seeing a dead corvid).

**Figure 2:**
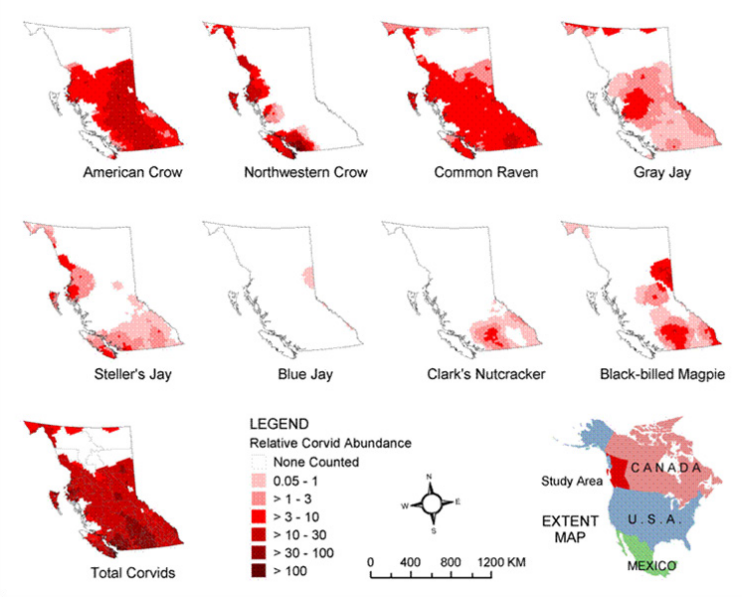
Relative corvid abundance by species in British Columbia from the North American breeding bird survey.

The relative density of corvids in each LHA was estimated using data from the BBS[12]. BBS routes consist of 50 stops spaced 0.8 km apart. Between May 28 and July 7 every year, volunteers record the number of individual bird species heard or seen within 0.4 km of each stop on these routes during three minutes of observation. Relative bird abundance map data, based on average counts from 1994–2003, in GIS raster format (~21 × 21 km cell size) were obtained from the United States Geological Survey[13]. These were based on inverse distance weighted surface interpolations performed on the BBS observation points[14]. Datasets for corvid species found in BC – American Crow (*Corvus brachyrhynchos*), Northwestern Crow (*Corvus caurinus*), Common Raven (*Corvus corax*), Gray Jay (*Perisoreus canadensis*), Steller's Jay (*Cyanocitta stelleri*), Blue Jay (*Cyanocitta cristata*), Clark's Nutcracker (*Nucifraga Columbiana*), and Black-billed Magpie (*Pica hudsonia*) – were imported into ArcGIS 8.3 (Environmental Systems Research Institute, Inc., Redlands, CA) for analysis. The Spatial Analyst extension of ArcGIS was used to generate a total corvid abundance data layer by adding the values of the individual corvid species (Figure 2), and neighbourhood statistics were used to summarize the mean values of the predicted numbers of corvids sighted for each LHA. This mean corvid count was used as an estimate of relative corvid density in each LHA.

The human population density of each LHA was calculated by dividing the population of the LHA by the LHA's geographic area. Population data were obtained from Population Extrapolation for Organizational Planning with Less Error (P.E.O.P.L.E.) Projection 29 [15]; and geographic data were obtained from the BC Ministry of Health Services.

**Figure 3:**
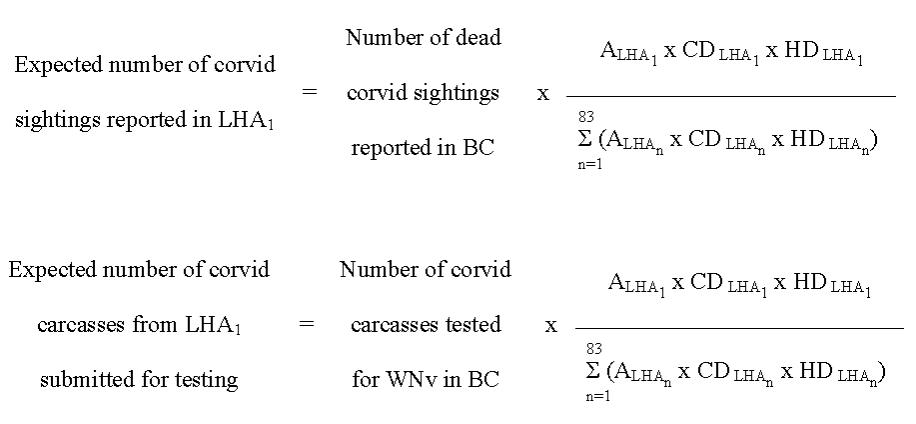
**Formulas used to calculate the expected values for corvid indicators in each Local Health Area**. LHA = Local Health Area; A = Geographic area; CD = Relative corvid density; HD = Human population density; WNv = West Nile virus.

The numbers of expected dead corvid sightings reported and corvids submitted for WNv testing by each LHA were calculated using the formulas outlined in Figure 3. The numbers of corvids sighted and tested from each LHA were compared to the expected numbers using the exact Poisson distribution and a significance level of 0.05.

## Competing interests

The author(s) declare that they have no competing interests.

## Authors' contributions

All authors participated in the design of the evaluation, were involved in critically revising the manuscript and read and approved the final manuscript. STD conducted the statistical analysis and drafted the manuscript. SM conducted the geographic analysis.

## Supplementary Material

Additional file 1 Numbers of corvids sighted and tested for West Nile virus. The data provided display the relative corvid density, area, population density, expected numbers of corvids sighted and tested, number of corvids sighted and number of corvids tested for each of the 83 Local Health Areas in British Columbia. Click here for file
