# Supplementary material for: A bird's eye view: using geographic analysis to evaluate the representativeness of corvid indicators for West Nile virus surveillance
Source: Int J Health Geogr. 2007 Jan 30;6:3. doi: 10.1186/1476-072X-6-3 (PMC1796857; doi:10.1186/1476-072X-6-3)
Supplement: Additional file 1 — Numbers of corvids sighted and tested for West Nile virus. The data provided display the relative corvid density, area, population density, expected numbers of corvids sighted and tested, number of corvids sighted and number of corvids tested for each of the 83 Local Health Areas in British Columbia. [file 1476-072X-6-3-S1.DOC]

**Numbers of corvids sighted and tested for West Nile virus**

| **Local Health Area Information** | | | | **Corvid Sightings** | | | **Corvids Tested** | | |
| --- | --- | --- | --- | --- | --- | --- | --- | --- | --- |
| **Local Health Area** | **Relative Corvid Density** | **Area**  **(km^2^)** | **Population**  **Density**  **(pop/km^2^)** | **Number Sighted** | **Number**  **Expected** | | **Number**  **Tested** | **Number**  **Expected** | |
| **Interior: East Kootenay** |  |  |  |  |  |  |  |  |  |
| Fernie | 19.0 | 8059 | 1.9 | 1 | 2 |  | 10 | 2 | A |
| Cranbrook | 15.3 | 4429 | 5.8 | 2 | 3 |  | 14 | 3 | A |
| Kimberley | 14.9 | 4361 | 2.0 | 0 | 1 |  | 2 | 1 |  |
| Windermere | 18.9 | 10974 | 0.9 | 0 | 1 |  | 6 | 1 | A |
| Creston | 16.5 | 3788 | 3.4 | 1 | 2 |  | 6 | 2 | A |
| Golden | 12.8 | 13350 | 0.6 | 1 | 1 |  | 3 | 1 | A |
| **Interior: Kootenay Boundary** |  |  |  |  |  |  |  |  |  |
| Kootenay Lake | 16.4 | 6536 | 0.5 | 0 | 0 |  | 0 | 0 |  |
| Nelson | 25.1 | 4794 | 5.2 | 2 | 5 |  | 15 | 5 | A |
| Castlegar | 33.1 | 1945 | 6.9 | 1 | 3 |  | 10 | 4 | A |
| Arrow Lakes | 24.8 | 7442 | 0.7 | 0 | 1 |  | 0 | 1 |  |
| Trail | 27.8 | 1137 | 18.1 | 0 | 4 | B | 8 | 5 |  |
| Grand Forks | 37.0 | 2686 | 3.4 | 2 | 2 |  | 9 | 3 | A |
| Kettle Valley | 38.9 | 4342 | 0.8 | 0 | 1 |  | 2 | 1 |  |
| **Interior: Okanagan** |  |  |  |  |  |  |  |  |  |
| Southern Okanagan | 38.6 | 1314 | 14.0 | 12 | 5 | A | 2 | 6 |  |
| Penticton | 39.2 | 1558 | 26.0 | 14 | 12 |  | 21 | 13 | A |
| Keremeos | 32.7 | 2485 | 2.0 | 3 | 1 | A | 2 | 1 |  |
| Princeton | 29.1 | 4815 | 1.0 | 2 | 1 |  | 0 | 1 |  |
| Armstrong-Spallumcheen | 36.9 | 261 | 38.5 | 5 | 3 |  | 3 | 3 |  |
| Vernon | 34.5 | 5555 | 10.8 | 22 | 15 | A | 19 | 17 |  |
| Central Okanagan | 38.2 | 2942 | 54.7 | 83 | 45 | A | 89 | 50 | A |
| Summerland | 37.6 | 624 | 18.7 | 2 | 3 |  | 2 | 4 |  |
| Enderby | 30.2 | 1861 | 3.9 | 4 | 2 |  | 1 | 2 |  |
| **Interior: Thompson Cariboo Shuswap** |  |  |  |  |  |  |  |  |  |
| Revelstoke | 14.0 | 9307 | 0.9 | 4 | 1 | A | 3 | 1 | A |
| Salmon Arm | 30.5 | 3113 | 10.3 | 13 | 7 | A | 24 | 8 | A |
| Kamloops | 32.5 | 16319 | 6.3 | 38 | 24 | A | 53 | 27 | A |
| 100 Mile House | 30.2 | 10721 | 1.3 | 5 | 3 |  | 7 | 4 |  |
| North Thompson | 18.7 | 12525 | 0.4 | 1 | 1 |  | 1 | 1 |  |
| Cariboo-Chilcotin | 21.2 | 44695 | 0.6 | 15 | 4 | A | 27 | 5 | A |
| Lillooet | 24.5 | 7464 | 0.6 | 3 | 1 | A | 1 | 1 |  |
| South Cariboo | 30.1 | 9474 | 0.8 | 3 | 2 |  | 0 | 2 |  |
| Merritt | 33.5 | 6591 | 1.7 | 1 | 3 |  | 1 | 3 |  |
| **Fraser: Fraser East** |  |  |  |  |  |  |  |  |  |
| Hope | 29.4 | 5281 | 1.6 | 8 | 2 | A | 4 | 2 |  |
| Chilliwack | 31.5 | 1314 | 56.8 | 36 | 17 | A | 37 | 19 | A |
| Abbotsford | 42.6 | 413 | 317.2 | 59 | 40 | A | 48 | 45 |  |
| Mission | 45.1 | 1439 | 26.4 | 13 | 12 |  | 6 | 14 | B |
| Agassiz-Harrison | 37.5 | 4080 | 2.1 | 15 | 2 | A | 5 | 3 |  |
| **Fraser: Fraser North** |  |  |  |  |  |  |  |  |  |
| New Westminster | 52.3 | 13 | 4491.7 | 26 | 23 |  | 26 | 25 |  |
| Burnaby | 52.3 | 90 | 2294.3 | 159 | 79 | A | 150 | 88 | A |
| Maple Ridge | 47.5 | 1450 | 61.7 | 28 | 31 |  | 34 | 34 |  |
| Coquitlam | 50.1 | 733 | 289.9 | 73 | 77 |  | 60 | 86 | B |
| **Fraser: Fraser South** |  |  |  |  |  |  |  |  |  |
| Langley | 48.6 | 323 | 365.0 | 44 | 42 |  | 57 | 46 | A |
| Surrey | 49.9 | 333 | 1262.1 | 80 | 152 | B | 97 | 169 | B |
| Delta | 51.2 | 186 | 547.1 | 34 | 38 |  | 30 | 42 | B |
| **Vancouver Coastal: Richmond** |  |  |  |  |  |  |  |  |  |
| Richmond | 53.8 | 124 | 1421.3 | 28 | 69 | B | 68 | 77 |  |
| **Vancouver Coastal: Vancouver** |  |  |  |  |  |  |  |  |  |
| Vancouver | 54.9 | 131 | 4425.9 | 139 | 232 | B | 209 | 258 | B |
| **Vancouver Coastal: North Shore/Coast Garibaldi** |  |  |  |  |  |  |  |  |  |
| North Vancouver | 53.4 | 398 | 342.5 | 130 | 53 | A | 34 | 59 | B |
| West Vancouver-Bowen Island | 61.0 | 232 | 215.5 | 22 | 22 |  | 31 | 25 |  |
| Sunshine Coast | 39.6 | 4029 | 6.8 | 3 | 8 | B | 5 | 9 |  |
| Powell River | 21.6 | 5254 | 3.8 | 2 | 3 |  | 4 | 4 |  |
| Howe Sound | 35.4 | 9236 | 3.4 | 3 | 8 | B | 9 | 9 |  |
| Bella Coola Valley | 11.4 | 25613 | 0.1 | 0 | 0 |  | 0 | 0 |  |
| Central Coast | 1.3 | 10601 | 0.2 | 0 | 0 |  | 1 | 0 | A |
| **Northern: Northwest** |  |  |  |  |  |  |  |  |  |
| Queen Charlotte | 16.2 | 9930 | 0.5 | 1 | 1 |  | 0 | 1 |  |
| Snow Country | 16.5 | 27815 | <0.1 | 0 | 0 |  | 0 | 0 |  |
| Prince Rupert | 28.3 | 6134 | 2.7 | 8 | 3 | A | 2 | 4 |  |
| Upper Skeena | 16.5 | 4456 | 1.3 | 0 | 1 |  | 1 | 1 |  |
| Smithers | 16.5 | 9827 | 1.8 | 2 | 2 |  | 0 | 2 |  |
| Kitimat | 12.5 | 19639 | 0.6 | 3 | 1 | A | 4 | 1 | A |
| Stikine | 4.3 | 132186 | 0.0 | 0 | 0 |  | 0 | 0 |  |
| Terrace | 23.7 | 13259 | 1.7 | 12 | 4 | A | 12 | 4 | A |
| Nisga'a | 21.8 | 5049 | 0.4 | 0 | 0 |  | 0 | 0 |  |
| Telegraph Creek | 5.5 | 23778 | <0.1 | 0 | 0 |  | 0 | 0 |  |
| **Northern: Northern Interior** |  |  |  |  |  |  |  |  |  |
| Quesnel | 23.4 | 23732 | 1.1 | 5 | 4 |  | 5 | 5 |  |
| Burns Lake | 14.8 | 25764 | 0.3 | 2 | 1 |  | 1 | 1 |  |
| Nechako | 14.7 | 42532 | 0.4 | 5 | 2 | A | 8 | 2 | A |
| Prince George | 15.1 | 76216 | 1.3 | 5 | 11 | B | 22 | 12 | A |
| **Northern: Northeast** |  |  |  |  |  |  |  |  |  |
| Peace River South | 23.3 | 27607 | 1.0 | 2 | 5 |  | 11 | 5 | A |
| Peace River North | 8.0 | 68766 | 0.5 | 2 | 2 |  | 7 | 2 | A |
| Fort Nelson | 0.6 | 89040 | 0.1 | 0 | 0 |  | 0 | 0 |  |
| **Vancouver Island: South** |  |  |  |  |  |  |  |  |  |
| Greater Victoria | 52.6 | 113 | 1847.0 | 39 | 80 | B | 37 | 88 | B |
| Sooke | 38.1 | 1744 | 32.2 | 2 | 16 | B | 5 | 17 | B |
| Saanich | 51.7 | 151 | 406.8 | 18 | 23 |  | 4 | 26 | B |
| Gulf Islands | 45.7 | 352 | 41.0 | 4 | 5 |  | 2 | 5 |  |
| **Vancouver Island: Central** |  |  |  |  |  |  |  |  |  |
| Cowichan | 43.4 | 737 | 71.7 | 0 | 17 | B | 5 | 19 | B |
| Lake Cowichan | 31.0 | 2398 | 2.6 | 0 | 1 |  | 1 | 2 |  |
| Ladysmith | 36.1 | 443 | 37.5 | 2 | 4 |  | 4 | 5 |  |
| Nanaimo | 31.9 | 1289 | 73.6 | 11 | 22 | B | 18 | 24 |  |
| Qualicum | 24.0 | 848 | 48.7 | 22 | 7 | A | 0 | 8 | B |
| Alberni | 14.5 | 6809 | 4.6 | 1 | 3 |  | 2 | 4 |  |
| **Vancouver Island: North** |  |  |  |  |  |  |  |  |  |
| Courtenay | 15.6 | 1751 | 33.6 | 3 | 7 |  | 22 | 7 | A |
| Campbell River | 10.9 | 13634 | 2.9 | 0 | 3 | B | 5 | 3 |  |
| Vancouver Island West | 7.7 | 5705 | 0.4 | 0 | 0 |  | 0 | 0 |  |
| Vancouver Island North | 3.9 | 20896 | 0.7 | 1 | 0 | A | 3 | 0 | A |

1. The observed value is statistically significantly larger than the expected value (p<0.05).
2. The observed value is statistically significantly smaller than the expected value (p<0.05).
